# Lean methodology use and nurses’ perception in the context of health management: a scoping review

**DOI:** 10.1590/0034-7167-2025-0130

**Published:** 2025-11-03

**Authors:** Marlene Cristina dos Santos, Jucinei Araújo de Jesus, Alexandre Pazetto Balsanelli

**Affiliations:** I Universidade Federal de São Paulo. São Paulo, São Paulo, Brazil

**Keywords:** Nurses, Health Management, Total Quality Management, Lean Healthcare, Six Sigma, Enfermeiras, Gestão em Saúde, Gestão da Qualidade Total, Saúde Enxuta, Seis Sigma, Enfermeras, Gestión de la Salud, Gestión de Calidad Total, Atención Sanitaria Ajustada, Seis Sigma

## Abstract

**Objectives:**

to identify the use of the Lean methodology by nurses and their perceptions.

**Methods:**

scoping review. The data search was conducted in the National Library of Medicine (PubMed), Web of Science, Latin American and Caribbean Literature in Health Sciences (LILACS), and Scientific Electronic Library Online (SciELO) databases between September 2023 and May 2024. Primary and secondary studies, reviews, and randomized trials published in Portuguese, Spanish, or English, available in full with free remote access, with no deadline, were included.

**Results:**

of 1,346 studies, 15 were included. The Lean methodology was identified in emergency flows (n=3), service management (n=5), Clinical Inpatient Units (n=3), Surgical Center (n=1), Intensive Care Units (n=1), Oncology (n=1), and Home Care Units (n=1).

**Conclusions:**

nurses use the Lean methodology, demonstrating satisfaction in most studies. A negative influence was observed related to the lack of resources for the use of the Lean methodology in nursing processes.

## RESUMO

**Objetivos:**

identificar a utilização da metodologia Lean por enfermeiros e suas percepções.

**Métodos:**

revisão de escopo. A busca dos dados foi feita nas bases *National Library of Medicine* (PubMed), *Web of Science*, Literatura Latino-Americana e do Caribe em Ciências da Saúde (LILACS) e *Scientific Electronic Library Online* (SciELO), entre setembro de 2023 e maio de 2024. Foram incluídos estudos primários, secundários, revisões e estudos randomizados, publicados em português, espanhol ou inglês, disponíveis na íntegra com acesso remoto gratuito, sem data limite.

**Resultados:**

de 1.346 estudos, 15 foram incluídos. A metodologia Lean foi identificada em fluxos de emergência (n=3), gestão de serviços (n=5), Unidades de Internação Clínica (n=3), Centro Cirúrgico (n=1), Unidades de Terapia Intensiva (n=1), Oncologia (n=1) e Unidades Domiciliares (n=1).

**Conclusão:**

os enfermeiros utilizam a metodologia Lean, demonstrando satisfação na maioria dos estudos. Observou-se influência negativa relacionada à falta de recursos para a utilização da metodologia Lean nos processos de enfermagem.

## RESUMEN

**Objetivos:**

identificar el uso de la metodología Lean por parte de enfermeras y sus percepciones.

**Métodos:**

revisión exploratoria. La búsqueda de datos se realizó en las bases de datos *National Library of Medicine* (PubMed), *Web of Science*, Literatura Latinoamericana y del Caribe en Ciencias de la Salud (LILACS) y *Scientific Electronic Library Online* (SciELO) entre septiembre de 2023 y mayo de 2024. Se incluyeron estudios primarios y secundarios, revisiones y ensayos aleatorizados publicados en portugués, español o inglés, disponibles en su totalidad con acceso remoto gratuito, sin fecha límite.

**Resultados:**

de 1.346 estudios, se incluyeron 15. La metodología Lean se identificó en flujos de emergencia (n = 3), gestión de servicios (n = 5), unidades de hospitalización clínica (n = 3), centro quirúrgico (n = 1), unidades de cuidados intensivos (n = 1), oncología (n = 1) y unidades de atención domiciliaria (n = 1).

**Conclusiones:**

las enfermeras utilizan la metodología Lean, demostrando satisfacción en la mayoría de los estudios. Se observó una influencia negativa relacionada con la falta de recursos para la utilización de la metodología Lean en los procesos de enfermería.

## INTRODUCTION

Lean thinking in healthcare took place in a structured and systematic manner in 2006. That year, the Lean Enterprise Academy (LEA), a non-profit organization in Great Britain focused on Lean thinking study and dissemination, organized the first congress on the application of Lean principles in healthcare services. Lean methodology in healthcare is a management approach that aims to improve the efficiency, quality and safety of healthcare services, reducing waste and increasing value for customers, becoming an increasingly relevant practice in healthcare service management throughout the world^(1,2)^.

Healthcare organizations are adopting Lean thinking as a strategy to offer better care in several countries, including the United States (ThedaCare (Wiscosin), Virginia Mason Medical Center (Seattle) and Martin Health Systems (Florida)), Sweden (Astrid Lindgren Children’s Hospital), the United Kingdom (Royal Bolton Hospital), and Australia (Flinders Medical Centre)^(1-7)^. Lean methodology provides organizations with conditions to increase the quality of patient care by reducing errors and waiting times. Furthermore, Lean is an approach that can support healthcare professionals, removing obstacles and allowing them to optimize time in providing care^(8)^.

Lean methodology in healthcare is still little explored, and studies are still being strengthened^(9)^, although articles on the use of lean tools have increased significantly in recent years^(10)^. In this context, nurses play a fundamental role, as they are on the front line of healthcare provision. The use of this methodology by these professionals can vary considerably. This occurs for several reasons, including training, experience, work environment and organizational culture^(11,12)^.

Therefore, it becomes important to understand how these professionals understand and use Lean methodology. A study carried out in Canada warned of the difficulties encountered by nurse managers in dealing with the responsibilities of adopting a new methodology that requires changes, with weaknesses and scarce resources in their setting. This concern is strengthened by the intensive nature of process improvement actions, which require complex, fast and dynamic responses to patients’ needs, which can generate a significant increase in work for nursing professionals in both the care and management spheres^(13)^.

Some difficulties are highlighted in this study as a possible justification for nurses’ resistance to adopting Lean methodology. Among these limitations are the limitation of training and preparation, associated with financial restrictions, difficulties in incorporating Lean as part of their leadership role in the face of workload of a complex and urgent nature in care settings, fragmented implementation of “easy to do” actions, but which were restricted to completing “checklists”, limiting an overview of the entire process^(13)^.

In this regard, this article aimed to identify whether nurses are using Lean methodology in their work processes and what their perceptions are regarding this use in the context of health management.

## OBJECTIVES

To identify Lean methodology use by nurses in the context of health management, considering these professionals’ perception

## METHODS

This is a scoping review, structured according to current JBI guidelines^(14)^. The choice for a scoping review was based on the need to map evidence on a given fact and identify existing gaps^(15)^. The use of selection criteria based on relevance to the phenomenon allows the scoping review to differentiate itself from other forms of reviews. The Preferred Reporting Items for Systematic reviews and Meta-Analyses extension for Scoping Reviews (PRISMA-ScR) checklist was used to conduct the study, present the results and prepare the final report, with a view to transparency and improving research methodological quality^(16)^.

Five stages were followed in this review: (1) research question identification; (2) study identification; (3) study selection; (4) data mapping; (5) grouping, synthesis and detailing and presentation of results. The review question was prepared using the PCC strategy, which includes the mnemonic acronyms as fundamental elements: P - Population, C - Concept and C - Context^(15)^, i.e., P= nurses, C= Lean methodology in healthcare use and perception, and C= health management. The objective was to answer the following question: do nurses use Lean methodology in healthcare in their work processes? What is professionals’ perception about the use of this methodology?

### Inclusion and exclusion criteria

Studies that addressed the topic, published in Portuguese, Spanish, or English, available in full via free remote access, with no date cut-off, were included. Primary and secondary studies, systematic, integrative, scoping reviews, and randomized studies that answered the research question, contemplating the use of Lean methodology and nurses’ perception, were included. Additional studies were included based on references cited in the articles extracted from primary sources (manual search). Partial research reports, editorials, response letters, narrative reviews, internet texts, books, abstracts published in event annals, articles not available in full in the databases and duplicate articles were excluded. The level of evidence was not considered an exclusion criterion.

### Research and search strategy for studies

Data collection was carried out between September 2023 and May 2024. The authors carried out searches in the National Library of Medicine (PubMed), Web of Science, Latin American and Caribbean Literature in Health Sciences (LILACS) and Scientific Electronic Library Online (SciELO) databases. The Medical Subject Headings (MeSH) (“Nurses”, “Health Management” and “Lean Healthcare”) and Descriptors in Health Sciences (DeCS) (“Nurses”, “Health Management”, “Total Quality Management”) descriptors were used, as described in Chart 1.

**Chart 1:** Mapping of search terms in DeCS and MeSH, São Paulo, São Paulo, Brazil, 2024

Term mapping - descriptors and synonyms - DeCS/MeSH				
PCC		Portuguese	English	Spanish
Participant	Nurses	*Enfermeiras e Enfermeiros*	Nurses	*Enfermeras y Enfermeros*
Concept	Lean methodology in healthcare vision, use and perception	*Gestão da Qualidade Total*	Total Quality Management	*Gestión de la Calidad Total*
		*Seis Sigma* *Lean*		
Context	Health Management	*Gestão em Saúde*	Health Management	*Gestión en Salud*

The descriptors were combined in different ways to expand the searches, in addition to using terminological variations and synonyms in the languages listed. The descriptors were combined using the Boolean operators AND (restrictive combination) and OR (additive combination). Among the keywords of the same acronym as the PCC strategy, OR was used. For the combination between different acronyms, AND was used. As this is a topic little explored in health, it was decided not to establish a date cut-off when searching for studies, as shown in Chart 2.

**Chart 2:** Database search strategy, São Paulo, São Paulo, Brazil, 2024

Database	Strategy used
PubMed	((nurse or nurses) and (health management) and ((total quality management)) or (Lean))
Web of Science	((*enfermeiros e enfermeiras*) AND (*gestão em saúde*) AND (*gestão de qualidade total* OR Lean healthcare))
LILACS	((*enfermeiros e enfermeiras*) AND (*gestão em saúde*) AND (*gestão de qualidade total*) OR (Lean healthcare))
SciELO	((*enfermeiros e enfermeiras*) AND (*gestão em saúde*) AND (*gestão de qualidade total*) OR (Lean healthcare))

### Study selection

The studies were selected by two authors (MCS and JAJ). Study selection according to title and abstract was carried out using the digital tool Rayyan QCR^(17)^. Subsequently, the two reviewers independently and blindly read the titles and abstracts, in order to reduce the possibility of interpretative bias. After reading and critically assessing the full texts of the pre-selected studies, applying the previously established inclusion and exclusion criteria, studies that did not meet the eligibility criteria were excluded. Disagreement among authors regarding eligibility was resolved by consensus between the three authors (MCS, JAJ and APB). Duplicate articles were also excluded from the sample.

### Data extraction and analysis

An instrument for data extraction was developed, and information mapping was carried out by two independent researchers. Data extraction and analysis were carried out by the main author (MCS) and verified by the author (JAJ).

Data analysis focused on identifying Lean methodology use by nurses and these professionals’ perception regarding the use of this methodology.

The following information was recorded for each article: article; authors; year of publication; study objective; results considering the use and nurses’ perception and country where the study was carried out. The protocol for this scoping review is registered with Open Science, under number DOI: 10.17605/OSF.IO/S9JKV.

**Figure 1:**
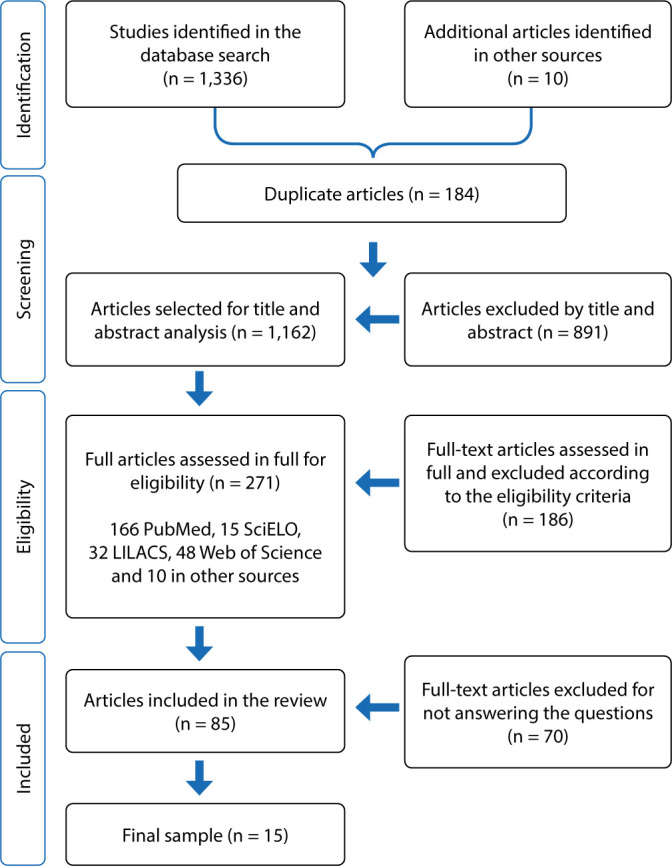
PRISMA flowchart (adapted) of the study selection process

Additional studies were included based on references cited in articles extracted from primary sources (manual search), as shown in Figure 1. Once this stage was completed, the studies were sent to Mendeley bibliography manager.

### Patient and/or public involvement

None.

## RESULTS

The search revealed 1,346 articles in the databases eligible for eligibility; 184 were discarded due to duplication; 1,162 studies underwent title and abstract analysis, and after this analysis 891 articles were excluded. Then 271 studies were selected for full reading. After reading for eligibility assessment, 85 studies were selected, and 70 were excluded for not answering the guiding questions, leaving 15 in the final sample. Chart 3 presents the results of included studies and their extracted information.

**Chart 3:** Studies that assessed the use and nurses’ perception regarding Lean methodology in healthcare

Article and Author Year of publication Citation number	Objective	Use and nurses’ perception	Country
*Pensamento Lean na saúde e enfermagem: revisão integrativa da literatura* Magalhães *et al*., 2016^(10)^	Highlight the scientific knowledge developed about Lean in the healthcare area, highlighting the impact and contributions to healthcare and nursing care.	Lean thinking use had a transformative effect on care and organizational aspects, promoting advantages in terms of quality, safety and efficiency of patient-focused healthcare and nursing.	Brazil
Easier and Faster Is Not Always Better: Grounded Theory of the Impact of Large-Scale System Transformation on the Clinical Work of Emergency Medicine Nurses and Physicians Zibrowski *et al*., 2018^(18)^	Explore ways in which a Lean intervention can impact emergency room nurses’ and physicians’ clinical work.	Nurses identified indications of job disqualification and how the new progressive model of patient care had detrimental impacts on their physical, cognitive and emotional well-being.	Canada
Quality Improvement: Implementing Nurse Standard Work in Emergency Department Fast-Track Area to Reduce Patient Length of Stay Williams *et al*., 2022^(19)^	Create and maintain standard nursing work to support a Lean improvement project in an urban emergency department.	Reduction in the average length of stay in the service area. The time spent walking to obtain supplies was reduced for all nursing professionals, generating satisfaction among professionals.	USA
*O olhar de enfermeiros assistenciais frente a implantação do programa Lean nas emergências hospitalares* Santos *et al*., 2021^(20)^	Analyze the impact on Lean project implementation from clinical nurses’ perspective in emergency services.	The study showed the need to adapt the nursing dimension as well as guarantee the forecast and provision of medical-hospital materials for quality care, seeking to add value to patients as well as their satisfaction with care at the institution.	Brazil
*Construção e validação de instrumento para avaliação do* Lean Healthcare *em instituições de saúde* Fernandes *et al*., 2024^(21)^	Build and validate an instrument to assess Lean healthcare in healthcare institutions.	For nursing and health management, the instrument can be useful in different settings/sectors in identifying weaknesses that compromise the maintenance of results achieved in Lean healthcare implementation.	Brazil
*Gestão hospitalar e gerenciamento em enfermagem à luz da filosofia* lean healthcare Silva *et al*., 2019^(22)^	Reflect on hospital management and nursing management linked to the Lean healthcare philosophy.	Both hospitals and nursing staff can benefit from the application of Lean concepts, in order to avoid waste, add value to the service and provide greater quality of healthcare.	Brazil
Role of lean leadership in the lean maturity second-order problem-solving relationship: a mixed methods study Bijl A *et al*., 2019^(23)^	Investigate the relationship between Lean methodology adoption and problem-solving behavior in nursing staffs.	As nursing staff reaches higher levels of Lean maturity, they also demonstrate higher degrees of second-order problem-solving.	Netherlands
Nurse managers implementing the lean management system: A qualitative study in Western Canada Udod AS *et al*., 2020^(24)^	Explore the perceptions and experiences of nurse managers involved in implementing the Lean management system in a western Canadian province.	Lean methodology implementation had a largely negative influence on nurse managers’ leadership roles.	Canada
Releasing Operating Room Nursing Time to Care through the Reduction of Surgical Case Preparation Time: A Lean Six Sigma Pilot Study Egan *et al*., 2021^(25)^	Describe a pilot Lean intervention designed to free up nursing time for care in a perioperative environment.	55% reduction in total nursing time spent collecting and preparing materials for surgical cases, with a corresponding reduction in packaging waste.	Ireland
Lean interventions in healthcare: do they actually work? A systematic literature review Moraros J, Lemstra M, Nwankwo C, 2016^(26)^	Independently assess the effect of Lean thinking and Lean interventions on worker and patient satisfaction.	Lean interventions had no statistically significant association with patient satisfaction and health outcomes; however, there was a negative association with financial costs and nursing worker satisfaction.	Canada
Optimizing nursing time in a day care unit: Quality improvement using Lean Six Sigma methodology Davies, Lyons, Whyte, 2019^(27)^	Apply Lean methodology to improve the efficiency of a private hospital children’s unit and generate a positive impact on optimizing nursing time	Significant impact on optimizing nursing time for direct patient care. Nurses expressed a feeling of greater autonomy, responsibility and justice in their professional lives.	Ireland
Healthcare workers’ perceptions of lean: a context-sensitive, mixed methods study in three Swedish hospitals Holden RJ *et al*., 2015^(28)^	Assess how hospital workers’ perceptions of Lean methodology vary between contexts: hospitals, types of units and professional roles.	Almost a third of nurses reported that they noticed substantial improvements in workflow.	USA
Pursuing nurses’ work effectiveness and better hand-hygiene compliance in a intensive care unit (ICU) ward: application of lean methodologies Gregório *et al*., 2015^(29)^	Explore Lean methodology use to improve nurses’ work processes in an Intensive Care U.	Increase in the global hand hygiene rate to 63%.	Portugal
Improving Nurses’ Hand-off Process on Oncology Setting Using Lean Management Principles Ayaad *et al*., 2019^(30)^	Improve hand-off effectiveness between nurses in the oncology environment using Lean management principles.	Significant reduction in the duration of shift change and the incidence of events related to deviation from nursing practice post-intervention. Furthermore, the results showed that nurses’ satisfaction rate improved.	Jordan
Home before Hospital: a whole of system re-design project to improve rates of home-based dialysis therapy: Experience and outcomes over 8 years Tombocon *et al*., 2021^(31)^	Improve prevalence rates of home dialysis therapy.	Improved nurse-led education. Reduction in time between referral from the nephrologist to the extension and the first contact with patient by the extension staff (<7 days in 100% of cases).	Australia

### Characteristics of selected studies

Studies were carried out between 2015 and 2024, and in 2017 and 2023 there was no study found. The predominant year of publications was 2019 (n=4), followed by 2021 (n=3). The countries where the studies were produced were Brazil (n=4), Canada (n=3), United States (n=2), Ireland (n=2), with one article each for Australia, Jordan, the Netherlands and Portugal. The majority were published in English (n=11) and the remainder were in Portuguese. As for study design, three articles were literature reviews, and the others varied between intervention (n=2), experimental (n=2), mixed-methods (n=2), observational (n=2), qualitative (n =1), cross-sectional (n=1), descriptive (n=1) and methodological (n=1) studies. Of the 15 studies selected, eight (53.3%) were carried out at hospitals and the remainder in various healthcare services.

**Figure 2:**
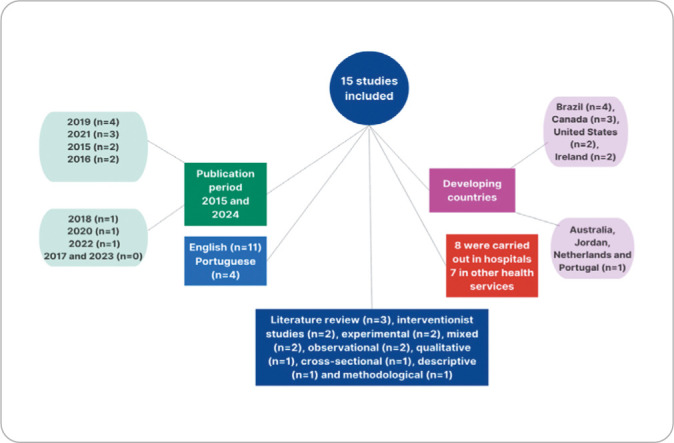
Mind map of the characteristics of selected articles

In order to present the information in a more detailed and visual manner, a mental map was created, contemplating the characteristics of selected articles, as shown in Figure 2.

### Evidence collected

Lean methodology application was identified in emergency flows (n=3), service management (n=5), care processes in Clinical Inpatient Units (n=3), Surgical Centers (n=1), Intensive Care Units (n=1), Oncology Units (n=1) and Home Units (n=1).

### Evidence application

In the 15 selected articles, it was possible to identify a variety in Lean philosophy use by nurses, applied in emergency flows^(18-20)^, service management^(10,21-24)^, care processes in Surgical Centers^(25)^, Clinical Inpatient Units^(26-28)^, Intensive Care Units^(29)^, Oncology Units^(30)^ and Home Units^(31)^. Nursing professionals’ perception was positive in most studies (n=12), demonstrating satisfaction with the improvements that occurred after adopting Lean philosophy, generating a reduction in rework, reduction in waiting time and optimization of flows. However, there was negative influence caused by a lack of planning and resources in Lean methodology implementation, which caused, in nurses’ view, work overload, and did not contribute to the desired improvements.

### Evidence assessment

Lean thinking use is on the rise among health research in the global context and permeates multiple care settings, but it is still in its infancy in nursing. Thus, it is observed that the articles were published especially from 2015 onwards in different journals and with different study designs, which allowed us to identify heterogeneity in the publication of the topic and in the distribution of the number of articles published among them.

### Results of changes identified

A variation was noticed in changes found in the articles analyzed. The management area was the most impacted, with positive results being observed^(10,21-23)^, followed by emergency^(19,20)^, Clinical Inpatient Units^(27,28)^, Surgical Centers^(25)^, Intensive Care Units^(29)^, Home Cares^(31)^ and Oncology Units^(30)^. All of these studies reported positive changes in their work processes.

## DISCUSSION

Studies have shown that Lean methodology use in emergency services made important contributions to reducing queues, waiting times and rework for the entire care staff, especially nursing, adding value to the care provided and reducing waste in healthcare institutions. However, it is clear that organizations must pay special attention to the adequate planning of the resources necessary to adopt Lean philosophy^(18-20)^.

Therefore, nurses need to be encouraged to identify and map bottlenecks in their work processes, including waste and rework, using tools that can improve flow and promote process restructuring, especially in emergency services, where time to care is always a critical factor and directly affects patients’ clinical outcome.

In management and nursing service management, Lean philosophy has also been used by nurses and has proven to be an important ally in improving processes, with the possibility of reducing costs and waste, with consequent generation of profits, appreciation of the service provided and achieving higher quality customer service, to which true value is attributed^(32)^.

In this setting, nurses play a fundamental role and can have their work process improved with the execution of Lean programs, as these professionals are the ones who are best able to decide what patients need to have their needs met, considering good clinical practices^(22,33)^. Even though the participation of nurses in scientific production related to this topic is not so effusive, they are considered a professional capable of leading a Lean transformation^(11)^. This result may be associated with the origin of Lean thinking in administration and engineering^(34)^.

Thus, it becomes clear that nurses need autonomy to make decisions about care and suggest improvements, creating a work environment that values their opinions and allows these professionals to apply Lean philosophy in their management practice, establishing regular cycles of discussions, alignments and planning, aiming to implement small improvements, but continuously, in nursing work processes.

Concerning the impact on Lean project implementation from clinical nurses’ perspective, problems such as increased workload and staffing can be highlighted^(20,35)^. Nursing staff, when providing care, faces limitations when it comes to inadequate and insufficient quantitative sizing of professionals and material resources available, making work exhausting, pointing to the need for adequate nursing staff sizing as well as forecasting and provision of medical-hospital materials for quality care, seeking to add value to patient care as well as their satisfaction with care at the institution^(20,31)^.

As for Lean functioning in the health context, the authors^(25,36)^ reached a negative perception of Lean in nurse involvement, focused on usefulness, patient care, time for patient care, workplace issues, supply availability, workload, stress, and patient safety. These results cause serious concerns, since worker involvement and contributions are essential for the success of Lean principles^(37)^, which contradicts other studies^(27)^.

Studies show that many nurses perceive that Lean methodology helps to optimize workflow, with clearer and better-defined processes, reducing time spent on unnecessary activities and thus allowing more time for patient care. However, there may be resistance if there is no clear communication about the benefits and if the change is not well managed and does not truly add value to these professionals. Lack of knowledge can also hinder nurses’ acceptance, since many may not have specific education or training in Lean, which can lead to inadequate implementation. Without the necessary knowledge and the correct sizing of resources, it will be difficult to apply lean tools effectively and sustainably.

Factors such as organizational culture, training, availability of resources, involvement of care teams and support from leadership are crucial to the success and sustainability of Lean projects in healthcare. When these barriers are not effectively overcome, institutions cannot maintain and evolve with the implementation of Lean philosophy, much less sustain its effective application in the long term^(20,24)^.

Furthermore, it becomes clear that organizations that wish to implement Lean methodology must make adequate resources available to finance the initiative, and it is essential to ensure that Lean tools are used in conjunction with the strategic elements of organizational readiness and leadership^(13)^. Leaders implementing Lean healthcare philosophy must recognize that, in the long term, some Lean practices may need to evolve from a focus on tools to a more holistic approach that changes the organization’s culture.

In this context, the role of a leader is even more important^(27)^. As nursing staff reaches higher levels of Lean maturity, they also demonstrate higher degrees of second-order problem-solving^(38)^. As such, evidence of the existence of a positive relationship between Lean maturity and second-order problem-solving is added^(23)^.

This suggests that nurses can become more adept at discovering and removing the root causes of organizational problems through the adoption of Lean methodology^(39,40)^. The reason is that Lean methodology helps nurses become familiar with and involved in identifying, analyzing and removing the root causes of problems. Lean leadership in wards has strong links with the Transformational Leadership Theory, such as awakening team spirit through motivation and inspiration are key activities in the inspirational motivation dimension^(41)^. Charismatic-inspirational leadership was also in evidence when Lean leaders excited others and, at the same time, actively participated in the Lean program^(42)^.

In an observational study^(28)^ with the aim of exploring Lean methodology use to improve nurses’ work processes in an Intensive Care Unit, the authors were able to report that a nurse can occupy an average of 16% of their working time using the information system, and the overall hand hygiene rate was 63%. Full adherence to hand hygiene would be equivalent to 13% of nursed’ workload. Furthermore, three processes were identified as the most relevant drivers: essential supply reorganization; optimized medication supply; and automatic data collection by monitoring equipment. The participating nurses defined the objectives of reducing the delivery time of supplies and establishing an inventory management system and continuously improving quality of care.

Moreover, Lean thinking is a management model that has emerged as a reference for achieving this quality of care combined with continuous improvement of processes^(43)^. Some studies highlighted that using Lean methodology can serve as support to help the health team to engage and take responsibility for significant improvements in quality of care, in addition to enabling targeted intervention strategies that have a significant impact on improving the results of services, teams and patients. These studies also highlight that nurses have an important and impactful role in the implementation and conduct of strategies that promote these improvements, including Lean philosophy, highlighting that the redesign and simplification of processes within a health setting can have a significant impact on optimizing the time of care teams to focus and dedicate themselves to direct patient care^(10,25,27)^.

### Study limitations

As a contribution to nursing, this review allows us to identify opportunities regarding Lean methodology in healthcare use and how it can be a great ally in improving nurses’ work processes, avoiding rework and favoring patient-centered care.

The result from this study provides a greater understanding of Lean thinking applicability related to nursing and may, in some way, contribute to improving patient care, especially in management and care professional satisfaction.

As a limiting factor of this study, it is worth noting that this review did not explore the costs and specific training of nurses involved in Lean healthcare implementation.

### Contributions to health, nursing, or public policy

It was observed that there is still a lack of studies on the topic as well as an absence of intervention studies with a view to testing hypotheses about the actions performed by nurses when using this methodology, providing opportunities for future research. Knowing more about Lean thinking, its principles and tools will enable changes in terms of seeing alternatives when carrying out the same process, with the aim of optimizing the result, reducing waste and adding value to the service provided.

## CONCLUSIONS

The study concluded that nursing professionals are using Lean methodology in healthcare more specifically for hospitals as a management tool to improve care processes, which has made it possible to reduce rework and optimize care flows. However, it was observed that there is a need to adapt human resources to adopt the methodology.

Concerning nurses’ perception, most studies reported a positive perception by professionals, evidenced by greater job satisfaction, reduced waste, greater autonomy and improvements in the environment. However, there was a negative influence related to the lack of planning of human resources and materials adequate for the implementation and maintenance of Lean philosophy in nursing processes, which deserves attention and further studies to deepen this aspect and broaden the vision of nurses’ satisfaction with the implementation of Lean philosophy.

Nurses’ perception of Lean methodology in healthcare is a crucial aspect for its successful implementation. Although there are advantages, such as improved efficiency and team engagement, and difficulties, such as resistance to change, lack of training and insufficient resources, it should not be underestimated. To maximize the benefits of Lean methodology, it is essential that healthcare institutions provide support, training and an environment that fosters innovation and collaboration.

## Data Availability

The research data are available within the article.
